# Optical, structural, and gamma shielding characteristics of bismuth-doped lithium borosilicate glass composite

**DOI:** 10.1038/s41598-025-23089-6

**Published:** 2025-10-31

**Authors:** A. Kh. Helmy, E. Salama, M. A. Azooz, F. H. Elbatal, M. A. Ouis, Elsayed E. Elshereafy

**Affiliations:** 1 Glass Research Department , National Research Centre , 33 EL Bohouth St., Dokki, Cairo, Egypt; 2 Basic Science Department Faculty of Engineering , The British University in Egypt (BUE), El-Sherouk City , Egypt; 3 Chemistry Department Faculty of Science , Menoufia University , Shebin El-Kom, Egypt

**Keywords:** Optical properties, Bismuth oxide, Lithium oxide, Borosilicate glasses, Radiation shielding, Chemistry, Materials science, Physics

## Abstract

This study investigates the optical, structural, and gamma radiation shielding properties of bismuth-doped lithium borosilicate glass composites. Glasses containing 0–20 mol% Bi₂O₃ were synthesized and analyzed using XRD, FTIR, density measurements, and UV–Vis spectroscopy. All samples exhibited an amorphous structure. The density increased markedly from 2.31 g/cm³ (0 mol% Bi₂O₃) to 4.59 g/cm³ (20 mol% Bi₂O₃), accompanied by a molar volume expansion from 27.33 to 31.02 cm³/mol. Optical studies revealed a progressive reduction in the band gap energy from 3.44 eV to 2.39 eV, while the Urbach energy increased from 0.216 eV to 0.488 eV, indicating enhanced structural disorder with Bi₂O₃ incorporation. Gamma attenuation analysis showed a significant improvement in shielding efficiency: at 0.662 MeV, the mass attenuation coefficient reached 9.83 × 10⁻² cm²/g with an effective atomic number up to 22.24, compared to only 7.76 × 10⁻² cm²/g for Portland concrete. Moreover, the half-value layer decreased with Bi₂O₃ loading, confirming improved attenuation performance. These results highlight that bismuth-doped lithium borosilicate glasses are promising lead-free materials for medical and nuclear radiation shielding applications.

## Introduction

The rise in using ionizing radiation for medical, industrial and nuclear purposes means there is now a major demand for strong and dependable radiation protection materials. Though effective, using lead as conventional shielding materials is challenging because it is toxic and very heavy, making it complicated to design with it^1,2^. For this reason, finding materials that minimize radiation as well as the issues already mentioned is now an important goal in radiation protection research.

An important difficulty is finding ways to reduce radiation but still keep the mechanical, thermal and chemical qualities of different materials high. Over the past few years, people have considered glass-based substances for shielding radiation because they are clear, simple to shape, don’t corrode and can host oxides of heavy metals to boost their properties^3–7^. What makes borate glasses attractive is their low melting point, good stability at high temperatures and strong capacity to incorporate heavy metal oxides into a single, stable structure without major crystallization. There is a large amount of literature looking at using metal oxides in borate glass to increase its shielding abilities^8^. For example, adding tungsten oxide (WO₃) to borate glasses contributed to increased effective atomic numbers and mass attenuation coefficients^9^. In a similar way, Kumar et al. (2019) looked at the effect of lead oxide (PbO) and barium oxide (BaO) and noticed improved performance as a gamma-ray shield^10,11^. Still, the problems lead causes and how it harms the environment mean it is necessary to find alternatives.

Rising Bi₂O₃ concentration in glasses of composition (60-x)B₂O₃–25Na₂O–13BaO–2Gd₂O₃–xBi₂O₃ (x = 0–20 mol%) reduces the glass transition temperature (T_g_), hardness, and band gap, while increasing non-bridging oxygens (NBOs) and the mass attenuation coefficient, indicating a less compact glass network^12^. Adding alkaline earth metal or metalloid oxides such as TeO₂ to 20BaO–15PbO₂–(65–x)B₂O₃–xTeO₂ glasses enhanced density, optimized optical properties, and significantly improved radiation shielding performance^13–17^.

Bismuth oxide is being seen as a promising material for shielding radiation because bismuth has Z = 83, is not poisonous, and attenuates gamma rays better than some similar substances. Synthesized barium borate glass samples containing different concentrations of Bi_2_O_3_ showed an improvement, particularly against low-energy gamma rays and X-rays with 20 mol% Bi_2_O_3_ optimal concentration^18,19^. The impact of increasing bismuth oxide content on thulium-doped borotellurite radiation shielding glass indicated that 20 mol% of Bi_2_O_3_ is the optimum glass sample for radiation shielding application^20^. A comprehensive analysis of silicate-based glasses with different Bi_2_O_3_ content shows alterations in the energy band gap values and optical properties^21^. A recent study examined the effects of substituting Li₂O with Ag_2_O in Li_2_O-MgO-Bi_2_O_3_-SiO_2_ glass, finding that Ag_2_O increased density, structural strength, insolubility, and radiation-shielding effectiveness. Glasses with higher Ag_2_O content showed improved mechanical, chemical, and attenuation properties, emerging as strong candidates for radiation protection. These findings provide a foundation for future glass development in advanced technological and shielding applications^22^. New approaches for these issues suggest mixing borate glasses with numerous heavy metal oxides or adjusting the structure and content of glass network formers and intermediates to improve both radiation protection and the glass’ characteristics^23^. Using computational and experimental methods has also been suggested to efficiently model and plan how glass systems behave structurally and when shielding against radiation^24^.

This study focuses on the preparation and characterization of a series of bismuth oxide-doped borate glasses with variable concentrations. The distinction of this work compared with similar studies lies in its focus on bismuth-doped lithium borosilicate glass composites and its comprehensive approach that integrates structural, optical, and gamma radiation shielding investigations. While many earlier studies concentrated on other heavy metal oxides in borate or silicate glasses or relied mainly on theoretical models, this research combines experimental measurements with theoretical comparisons, particularly of the mass attenuation coefficient. It further demonstrates the influence of Bi_2_O₃ concentration on HVL, density, and shielding performance, showing that thinner samples can provide effective radiation protection due to the high atomic number and density of bismuth. Overall, the novelty of this study is its systematic experimental–theoretical evaluation of Bi₂O₃-doped lithium borosilicate glasses, offering a broader and more integrated contribution than previous works.

## Materials and methods

### Preparation of the glass samples

**Fig. 1:**
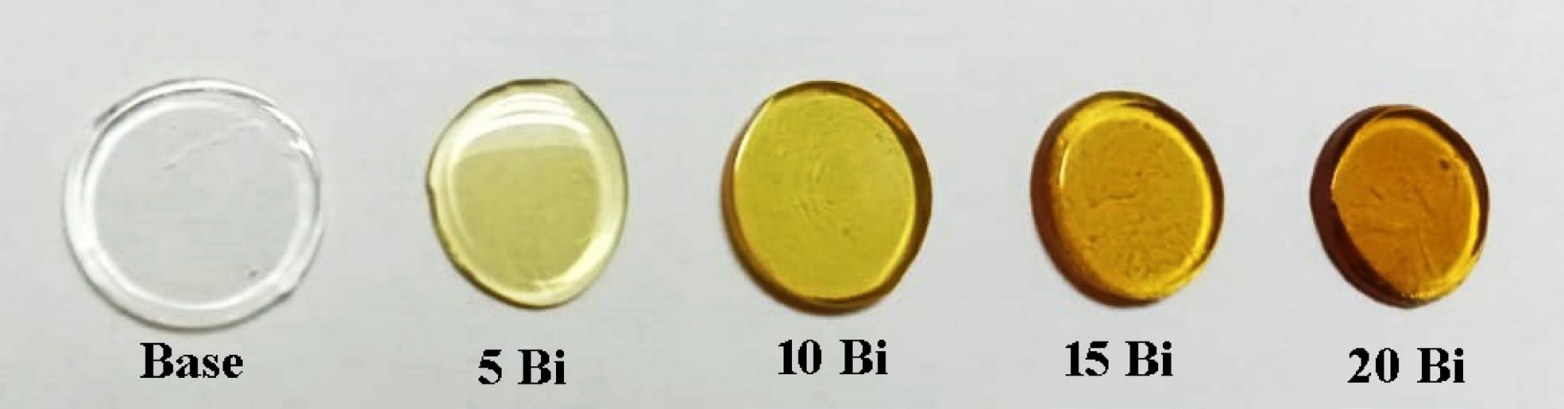
The photographs of the fabricated glass samples.

The parent glasses were prepared using laboratory-grade chemicals with high purity of 99.9% (Sigma Aldrich Company) for orthoboric acid (H_3_BO_3_) as a source of B_2_O_3_, calcium carbonate (CaCO₃) as a source of CaO, sodium carbonate (Na₂CO₃) as a source of Na_2_O, and lithium fluoride (LiF) was used as such. Silicon oxide (SiO₂) from Fisher Chemicals Company, with a purity of 99.89% and bismuth oxide (Bi_2_O_3_) from Alfa Aesar Company, with a purity of 99% were used as such. The detailed chemical compositions of the prepared glasses are listed in Table 1. The melting quenching technique was used for preparing glass batches. The photographs of the fabricated glass samples are shown in Fig. 1. Precisely weighed batches of these materials were melted in covered alumina crucibles at 1250 ± 10 °C for 90 min using a silicon carbide (SiC)-heated furnace (Vecstar, UK). To ensure uniformity, the molten mixtures were stirred multiple times before being poured into preheated stainless steel molds of the desired dimensions. The resulting glass samples were then immediately transferred to an annealing muffle set at 400 °C, where they were held for 1 h. Following this, the furnace was turned off, allowing the samples to cool gradually to room temperature at a controlled rate of 30 °C per hour.

### Sample characterization

X-ray diffraction (XRD) analysis was carried out on powdered samples at room temperature using an Empyrean diffractometer (Panalytical, Almelo, The Netherlands). The system employed a Cu X-ray tube operating at 45 kV and 30 mA, with a Ni filter to remove K_β_ radiation and a Pixcel3D detector. Diffraction patterns were recorded in step-scan mode over a 2θ range of 5° to 80°, with a step size of 0.03° and a counting time of 20 s per step.

The structural characterization of the building units within the prepared glasses was performed by recording their Fourier Transform Infrared (FTIR) absorption spectra. Measurements were conducted using a Bruker VERTEX 80 FTIR spectrometer (Germany) equipped with a Platinum Diamond ATR accessory, featuring a diamond disk as the internal reflector. Spectra were collected across the 4000–400 cm⁻¹ range, with a spectral resolution of 4 cm⁻¹ and a refractive index of 2.4.

Optical (UV–Visible) absorption spectra of the polished glass samples, all having a uniform thickness of 2 ± 0.1 mm, were obtained at room temperature using a Jasco V-570 recording spectrophotometer over the wavelength range of 190–1200 nm. Each specimen was measured twice to ensure the reproducibility and accuracy of the absorption peak data.

The gamma-ray attenuation characteristics of the prepared glass samples were evaluated using a “2 × 2” NaI(Tl) scintillation detector from Teledyne Isotopes (Alabama, USA), under optimized geometrical conditions. The system achieved an energy resolution of 8% at 0.662 MeV. Mass attenuation coefficients (µm) and exposure build-up factors (EBF) were determined across the photon energy range of 0.015–15 MeV and for sample thicknesses up to 40 mean free paths (mfp). Calculations were based on the Lambert-Beer law and the Geometric Progression (G-P) fitting method. Additionally, theoretical radiation shielding parameters were computed using the Phy-X/PSD software, referencing data from the National Institute of Standards and Technology (NIST)^25^. The effective atomic number (Z_eff_) of each glass composition was also calculated, taking into account effective atomic and electronic cross-sections, elemental composition, atomic weights, and fractional abundances.

**Table 1:** Code and chemical composition of the prepared glass samples.

Sample code	Composition (mol%)						Density (g/cm^3^)
	SiO_2_	B_2_O_3_	CaO	Na_2_O	LiF	Bi_2_O_3_	
Base	30	50	5	10	5	0	2.31
5Bi	30	45	5	10	5	5	2.98
10Bi	30	40	5	10	5	10	3.56
15Bi	30	35	5	10	5	15	4.09
20Bi	30	30	5	10	5	20	4.59

### Mass density and molar volume

The experimental density (ρ) of the glass samples was determined using the Archimedes principle, where each sample was first weighed in air and then submerged in xylene. The density was calculated using the following equation:

1 $$\:{\uprho\:}=\frac{{\text{W}}_{a}}{{\text{W}}_{a}-{\text{W}}_{b}}\:{{\uprho\:}}_{b}$$

where W_a_​ represents the weight of the sample in air, W_b_​ is its weight when immersed in xylene, and ρ_b_​ is the density of xylene, taken as 0.865 g/cm³. Subsequently, the molar volume (V_m_) was determined by dividing the molar mass (M) of the glass composition by its measured density (ρ).

## Results and discussions

### XRD

**Fig. 2:**
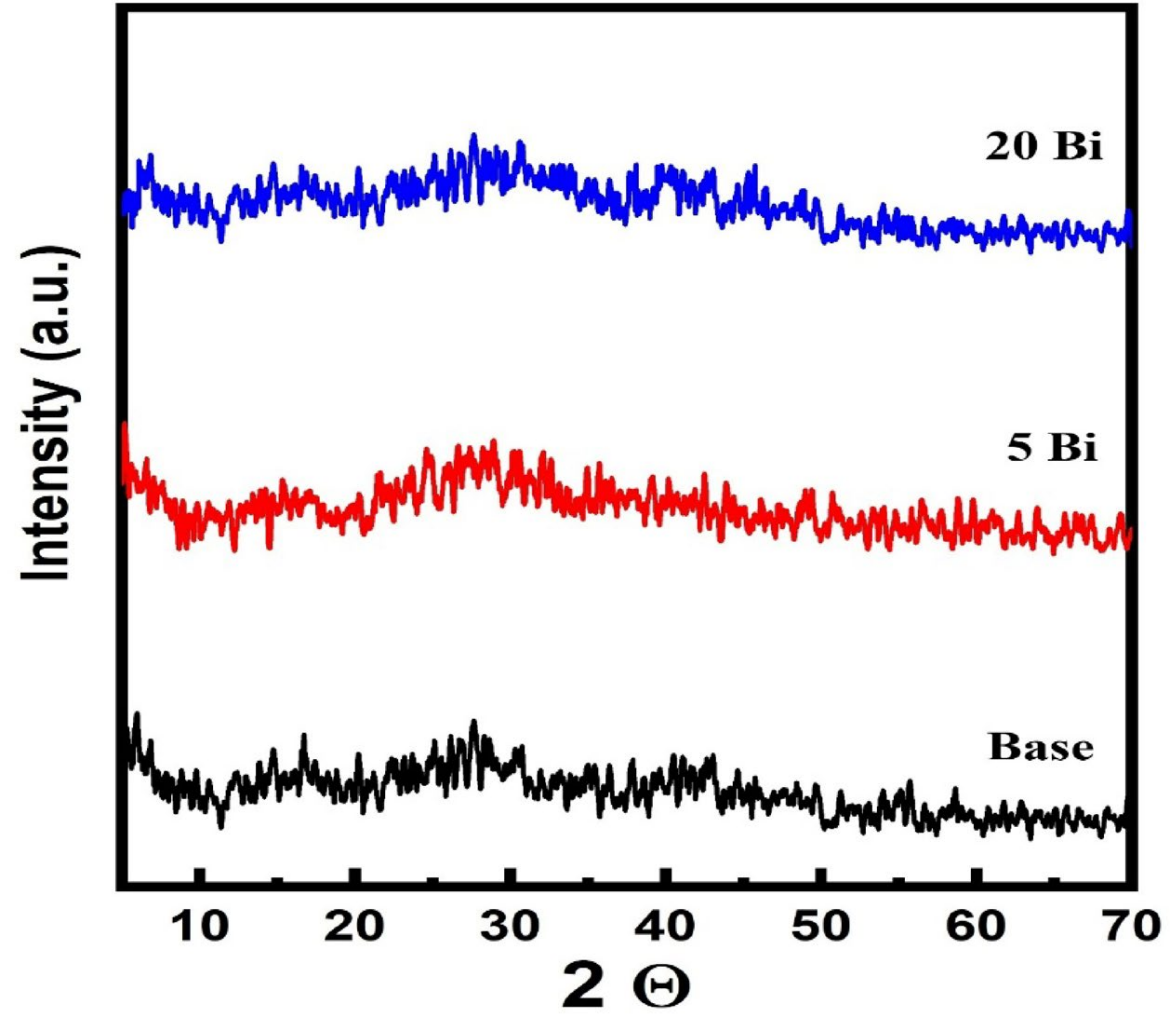
X-ray diffraction patterns for some selected glass samples (Base, 5 Bi, and 20 Bi).

The patterns in Fig. 2 show the XRD results of the prepared glass samples. The absence of sharp diffraction peaks indicates that the samples possess an amorphous structure. The broad variations in the patterns arise from X-ray scattering in the disordered glass, suggesting structural irregularities over a wide range. Consequently, the materials are confirmed to be non-crystalline and glassy. Typically, borate glasses exhibit a broad halo in their XRD patterns between 20° and 30°^26^.

### FTIR analysis

In this study, special attention was focused on the mid-infrared region (400–1600 cm⁻¹), where various vibrational modes associated with Bi–borosilicate glasses are observed, to elucidate the role of Bi₂O₃ in the prepared glass systems. The recorded spectra displayed multiple absorption bands across different spectral ranges, with minor shifts in both intensity and position. These bands often overlapped and broadened, making the direct interpretation of individual peaks challenging.

**Fig. 3:**
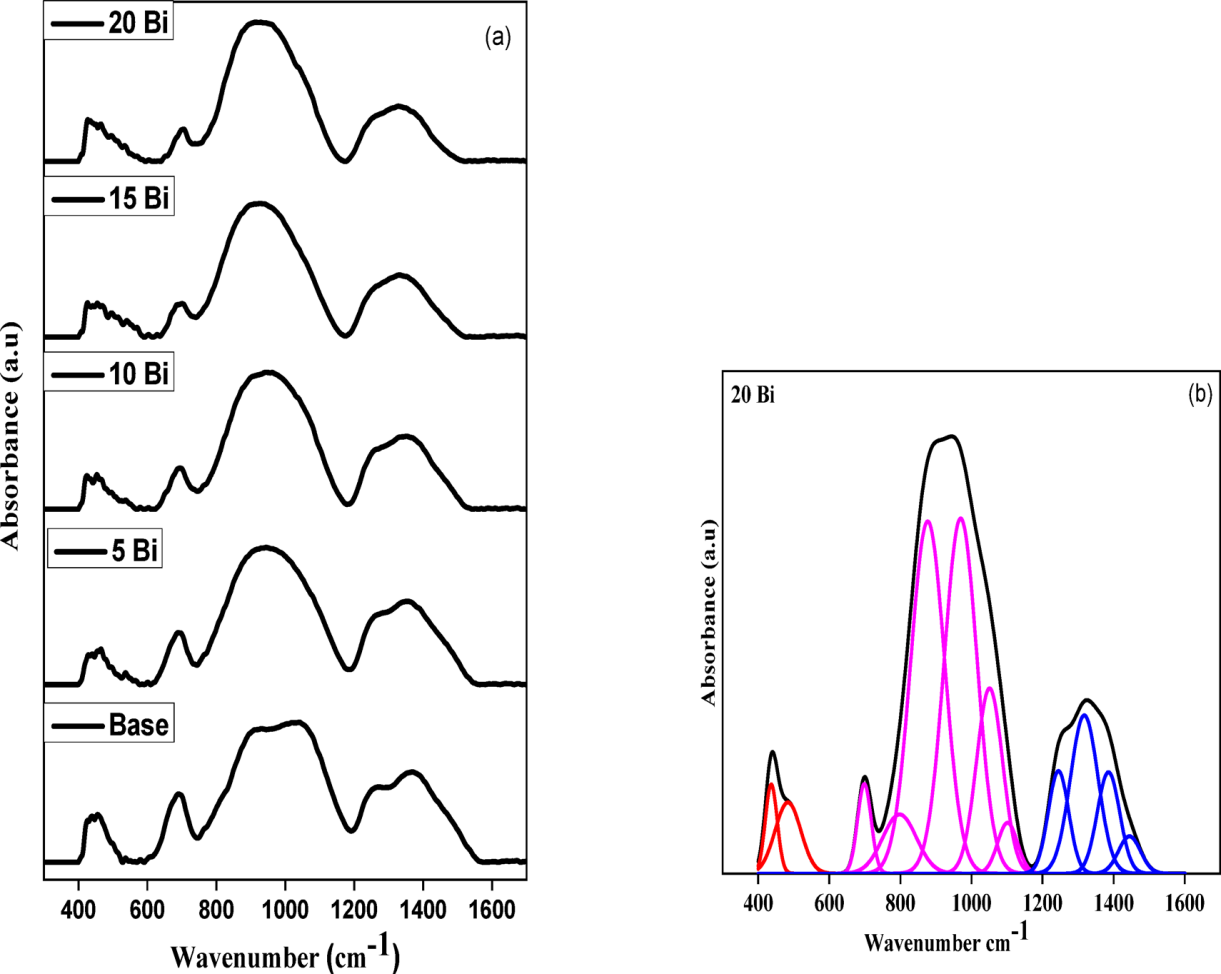
(a) FTIR spectra for the prepared glass samples (b) deconvoluted infrared spectra for 20 Bi glass sample, as a representative example.

To address this, a deconvolution procedure was applied to the FTIR spectra, separating the overlapping bands into distinct peaks for clearer identification. The original FTIR spectra of the prepared glass samples are shown in Fig. 3(a), while a representative set of deconvoluted peaks is presented in Fig. 3(b). The identified peak positions and their corresponding assignments are summarized in Table 2.

**Table 2:** Center and integrated area of all deconvoluted peaks for all samples.

Peak No.	Base		5 Bi		10 Bi		15 Bi		20 Bi	
	Center	Area	Center	Area	Center	Area	Center	Area	Center	Area
1	428	0.3748	429	0.265	426	0.2882	430	0.263	437	0.7311
2	461	1.228	464	0.6886	459	0.5664	476	1.491	484	1.274
3	687	2.6435	689	1.608	692	1.053	696	1.16	698	0.856
4	768	0.4133	804	1.642	777	0.652	783	1.094	797	1.406
5	822	2.2583	880	5.349	867	5.386	867	7.438	876	8.35
6	904	7.019	966	6.161	958	6.459	971	9.581	969	8.15
7	994	5.996	1035	2.62	1049	4.715	1054	1.689	1050	3.404
8	1082	7.502	1097	2.838	1118	0.496	1102	1.039	1101	0.712
9	1250	2.996	1250	1.463	1254	1.726	1243	1.225	1244	1.471
10	1367	7.1367	1355	6.72	1327	2.656	1310	3.101	1317	3.077
11	1473	1.121	1470	0.5899	1395	1.995	1385	2.373	1385	1.595
12					1460	0.628	1453	0.436	1444	0.577
**BO**_**4**_	22.775		18.61		17.056		19.747		20.616	
**BO**_**3**_	11.2537		8.7729		7.005		7.135		6.72	
**BO**_**4**_ **+ BO**_**3**_	34.0287		27.3829		24.061		26.882		27.336	
**N**_**4**_	0.669		0.68		0.709		0.735		0.754	

The absorption bands detected in the 400–600 cm⁻¹ region are primarily attributed to the vibrational modes of heavy metal oxides in the glass framework^27^. Specifically, the peaks near 484 cm⁻¹ and 437 cm⁻¹ correspond to the combined bending vibrations of Bi–O bonds in BiO₆ octahedral units within the borate network and the bending vibrations of Si–O–Si linkages in SiO₄ tetrahedra, respectively^28,29^. The band observed around 876 cm⁻¹ is associated with di-borate units and B–O–B linkages in the borate network^30^, while the peak near 698 cm⁻¹ reflects both B–O–B bending vibrations and the stretching modes of Bi–O bonds in BiO₃ units^29,31^.

In the spectral range of approximately 797–1100 cm⁻¹, several absorption bands appear, which can be categorized into two groups based on the vibrations of silicate and borate tetrahedral units^32–34^. Within the borosilicate network, the bands at 969 cm⁻¹ and 1101 cm⁻¹ correspond to the asymmetric stretching vibrations of Si–O–Si linkages and B–O–Si bonds, respectively. Additionally, the absorption bands at 876 cm⁻¹ and 1050 cm⁻¹ are associated with the stretching vibrations of BO₄ tetrahedral units^33–36^.

Finally, the absorption region between 1200 and 1500 cm⁻¹ was deconvoluted into four distinct bands at 1244, 1317, 1385, and 1444 cm⁻¹. These are typically assigned to the B–O stretching vibrations within triangular borate (BO₃) groups^37^.

The bands attributed to the borate network were utilized to interpret the infrared spectra obtained through the deconvolution process. The areas under the deconvoluted peaks were measured to calculate specific structural parameters, including the N₄ and non-bridging oxygen (NBO) ratios. The N₄ parameter, which offers essential information about the proportion of BO₄ units within the glass structure, was calculated using the following equation^33^:

2 $${\text{N}}_{4} = \frac{{{\text{The}}~{\text{area}}~{\text{of}}~{\text{BO}}_{4} }}{{{\text{Total}}~{\text{area}}~{\text{of}}~\left( {{\text{BO}}_{3} + ~{\text{BO}}_{4} } \right)}}$$

**Fig. 4:**
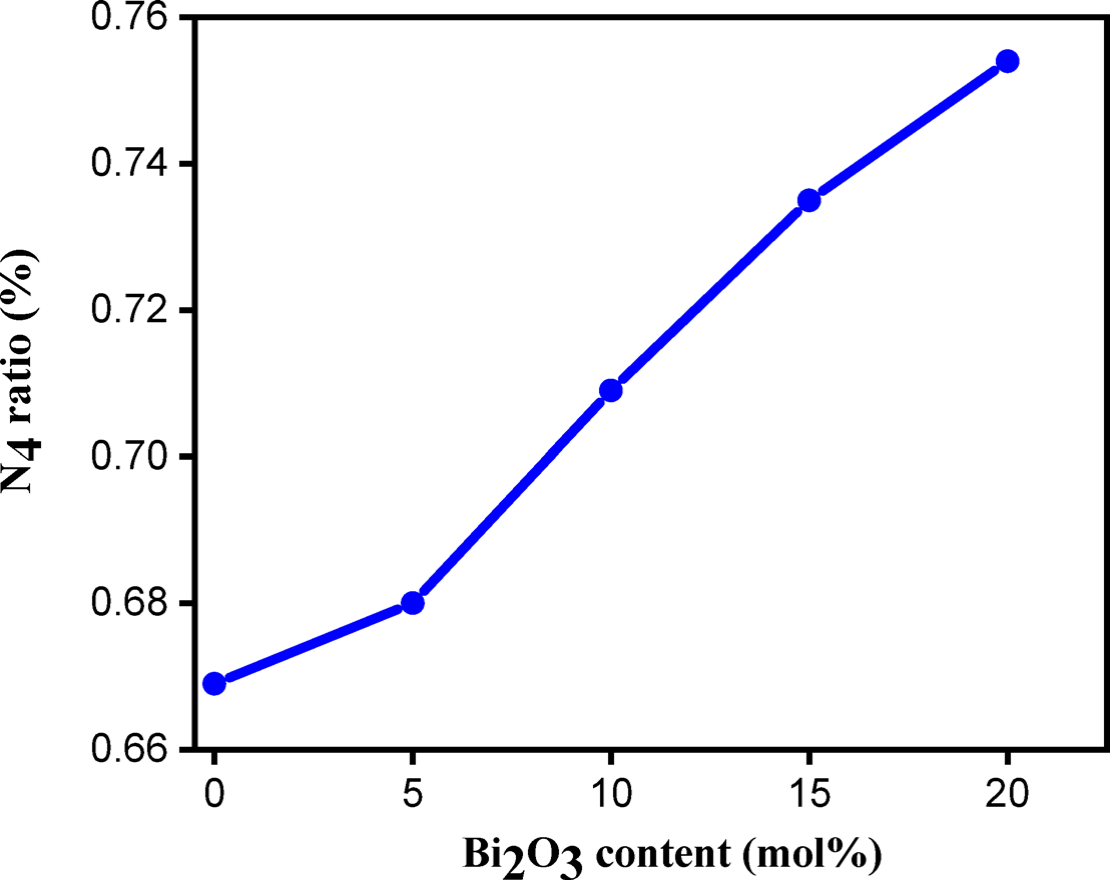
The dependence of *N*_4_ ratio on Bi_2_O_3_ content.

Figure 4 shows that the N₄ ratio increases with the rising Bi₂O₃ content. The incorporation of Bi ions into the glass matrix leads to the transformation of some BO₃ units into BO₄ units, resulting in an increased content of non-bridging oxygen (NBO) bonds^38^. A comparable trend has been observed in previous studies on bismuth-doped borosilicate glasses^39^. This observation is further supported by the reduction in optical band gap values and the corresponding rise in Urbach energy values as the Bi₂O₃ content increases.

### Mass density and molar volume

**Fig. 5:**
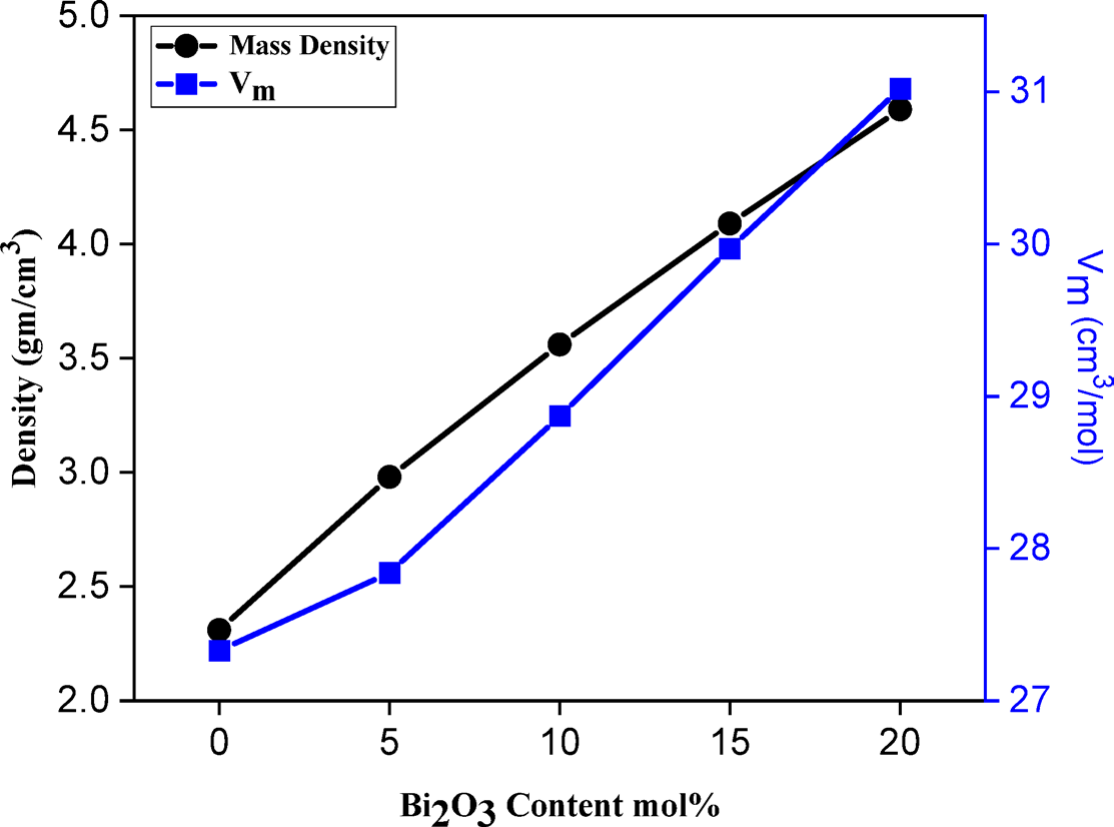
The relation between both mass density and molar volume of the prepared glass samples.

The mass density (ρ) and molar volume (Vm) of glass samples are important parameters for investigating structural changes. These properties are influenced by several factors, including coordination number, network structure, cross-link density, and interstitial spaces^40^. Figure 5 illustrates the relationship between ρ and Vm as a function of Bi₂O₃ content, while the corresponding values for the prepared glass compositions are listed in Table 3.

**Table 3:** Density (ρ) and molar volume (Vm) of the glass samples.

Sample	Density (*ρ*)	V_m_	M_wt_
Base	2.31	27.33	63.13
5 Bi	2.98	27.84	82.95
10 Bi	3.56	28.87	102.77
15 Bi	4.09	29.97	122.58
20 Bi	4.59	31.02	142.4

In general, the introduction of Bi₂O₃ into the glass network led to a significant increase in mass density, rising from 2.31 g/cm³ (0% mol Bi₂O₃) to 4.59 g/cm³ (20% mol Bi₂O₃), with an uncertainty of ± 0.005 g/cm³. This increase in ρ can be attributed to the substitution of lighter B ions (2.34 g/cm³) with heavier Bi ions (9.78 g/cm³). Moreover, replacing boron oxide with bismuth oxide increases the coordination number to six, compared with the three- or four-fold coordination typical of boron oxide, thereby enhancing the density^41^.

In the prepared glass samples, the molar volume (V_m_) also increased gradually with Bi₂O₃ content, from 27.33 cm³/mol at 0% Bi₂O₃ to 31.02 cm³/mol at the highest concentration. This expansion of V_m_ is likely due to the rise in non-bridging oxygen atoms (NBOs) with increasing Bi₂O₃ content, which opens up the tightly compacted borosilicate network. As a result, the glass structure becomes less compact, leading to a noticeable change in the internal network, consistent with predictions from IR analysis^42,43^.

### Optical characteristics

**Fig. 6:**
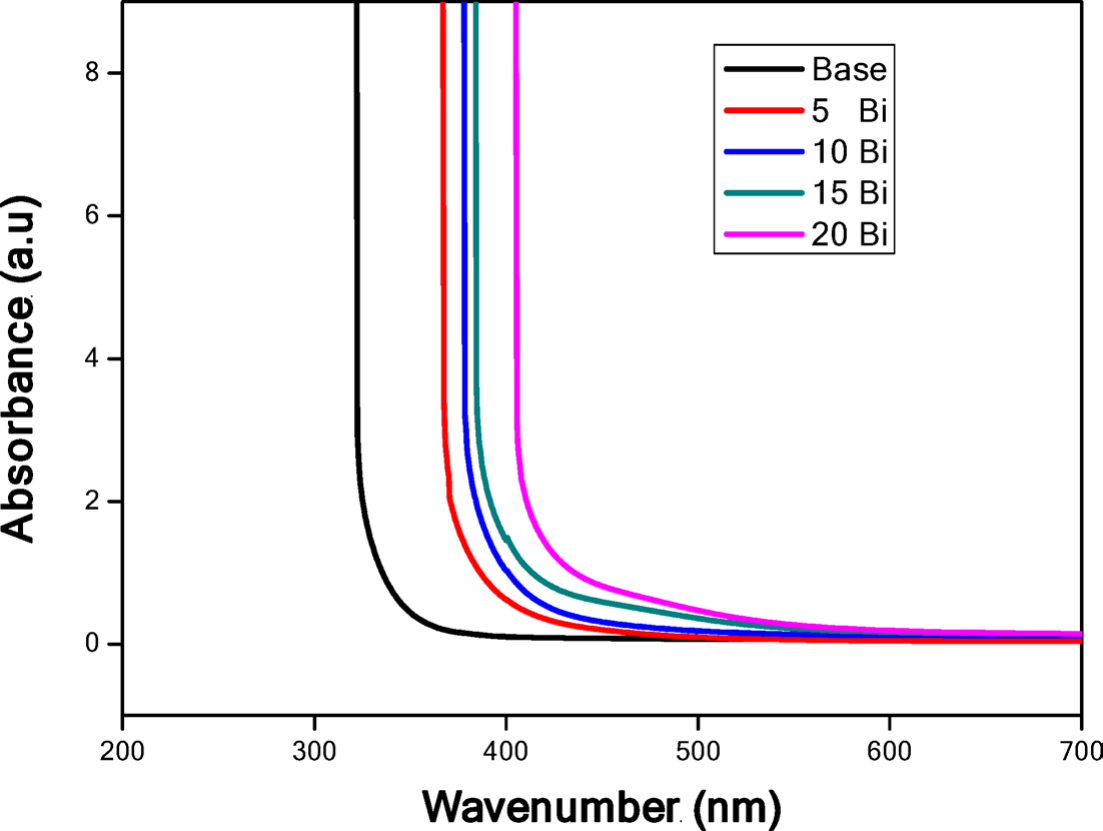
Optical absorption spectra for the prepared glass samples.

UV–visible spectroscopy is a powerful tool for investigating the optical properties of materials, including band gap and Urbach energies. Figure 6 presents the UV–visible diffuse absorbance spectra of the examined glass samples. As shown, all samples exhibit a similar spectral profile, with an absorption edge that progressively shifts toward longer wavelengths (red shift) as the Bi₂O₃ concentration increases in the glass matrix.

The absorption edge spans a broad wavelength range of 320–400 nm in the base glass, reflecting the amorphous nature of the prepared samples. Below 320 nm, the spectra display a saturation region, followed by a decrease in absorption beyond 320 nm, corresponding to valence-to-conduction band transitions. The degree of attenuation and curvature at the absorption edges is influenced by impurities and structural defects in the glass network. Notably, no optical transitions appear in the visible region, since such transitions generally arise from d–d electronic transitions, and this glass composition contains no d-orbital heavy metal oxides capable of producing them.

The optical spectra of non-crystalline materials typically exhibit three distinct regions: (i) a phonon-assisted constant absorption region, (ii) the Tauc region, corresponding to strong absorption due to inter-band electronic transitions, and (iii) the Urbach region, where the absorption coefficient shows an exponential dependence on photon energy^44^.

#### Band gap energy

**Fig. 7:**
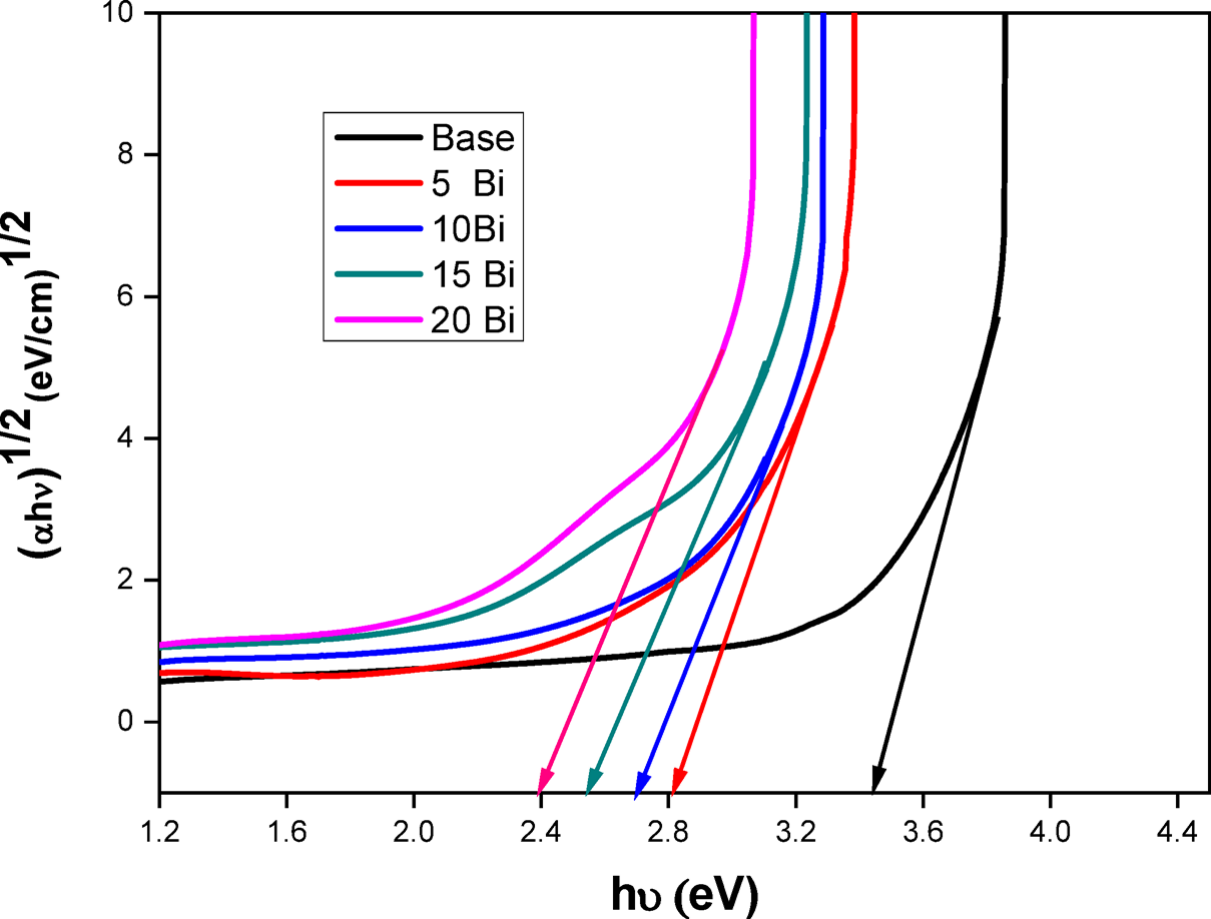
Taucs plot reveals the determination of band gap energy values for all produced glass samples.

The optical band gap represents the energy separation between the valence band maximum and the conduction band minimum. For amorphous materials, it can be determined using models based on the Tauc method, originally proposed by Davis and Mott, expressed by the following relation^45,46^:

3 $$\:{\upalpha\:}\text{h}{\upupsilon\:}\:=\text{q}\:(\:\text{h}{\upupsilon\:}\:-\:{\text{E}}_{\text{g}}{)}^{m}$$

where hυ is the photon energy, q is a constant, E_g_ is the optical band gap, m refers to an index that has different values (1/2 for direct and 2 for indirect allowed transitions), and *α* is the absorption coefficient. The origin in selecting the best value among them depends on the optical transitions as well as the material type^47,48^. When the condition (αhυ)^0.5^ is applied as Y-coordinate against photon energy (hυ) as X-coordinate, the band gap energy value is equal to the intersection of the first straight portion of the linear part of the spectrum with X-ordinate. Figure 7 shows the optical spectra for all prepared samples with determination of E_g_ Values. The band gap values are listed in Table 4 and noticed to decrease steadily with increasing the concentration of Bi_2_O_3_ from 3.44 eV for the Base sample (0 mol% Bi_2_O_3_) to 2.39 eV for the highest concentration (20 mol% Bi_2_O_3_).

**Table 4:** Band gap energy and Urbach tail of the prepared glass samples.

Bi_2_O_3_ (mol%)	Energy gap Eg (ev)	Urbach energy (ev)	Slope
Base	3.44	0.2164	4.62
5 Bi	2.81	0.32	3.12
10 Bi	2.70	0.365	2.74
15 Bi	2.55	0.456	2.19
20 Bi	2.39	0.488	2.05

As noted in the infrared analysis, increasing the Bi₂O₃ concentration promotes the formation of non-bridging oxygen (NBO) bonds within the glass structure. These NBOs introduce additional energy levels near the top of the valence band, facilitating the transition of valence electrons to these states. Consequently, the band gap energy decreases progressively with increasing dopant concentration^49,50^.

#### Urbach tail

In amorphous or poorly crystalline materials, the optical absorption edge exhibits an exponential relation between the absorption coefficient (α) and the photon energy (hυ), forming a characteristic tail^51^. The Urbach energy (EU), which represents the width of these band tails^52^, arises from structural disorder in the material. Networks with higher defect concentrations—such as non-bridging oxygens (NBOs) and dangling bonds—typically exhibit larger EU values. These defects introduce additional energy states within the bandgap, further confirming the amorphous structure of the glass samples.

To determine EU for our glass series, we use the simplified Urbach relation given in Eq. (4)^51^:

4 $$\:\text{L}\text{n}\:\left({\upalpha\:}\right)=\text{L}\text{n}\:\left(\text{k}\right)+\:\:\text{h}{\upupsilon\:}\:({\text{E}}_{\text{U}}{)}^{-1}\:$$

where k is a constant. By plotting hυ against Ln (α), the Urbach energy can be derived from the inverse slope of the linear region in the resulting graph. Higher E_U_ values indicate an increased likelihood of weak bonds transforming into defects, making Urbach energy a useful metric for defect concentration analysis.

**Fig. 8:**
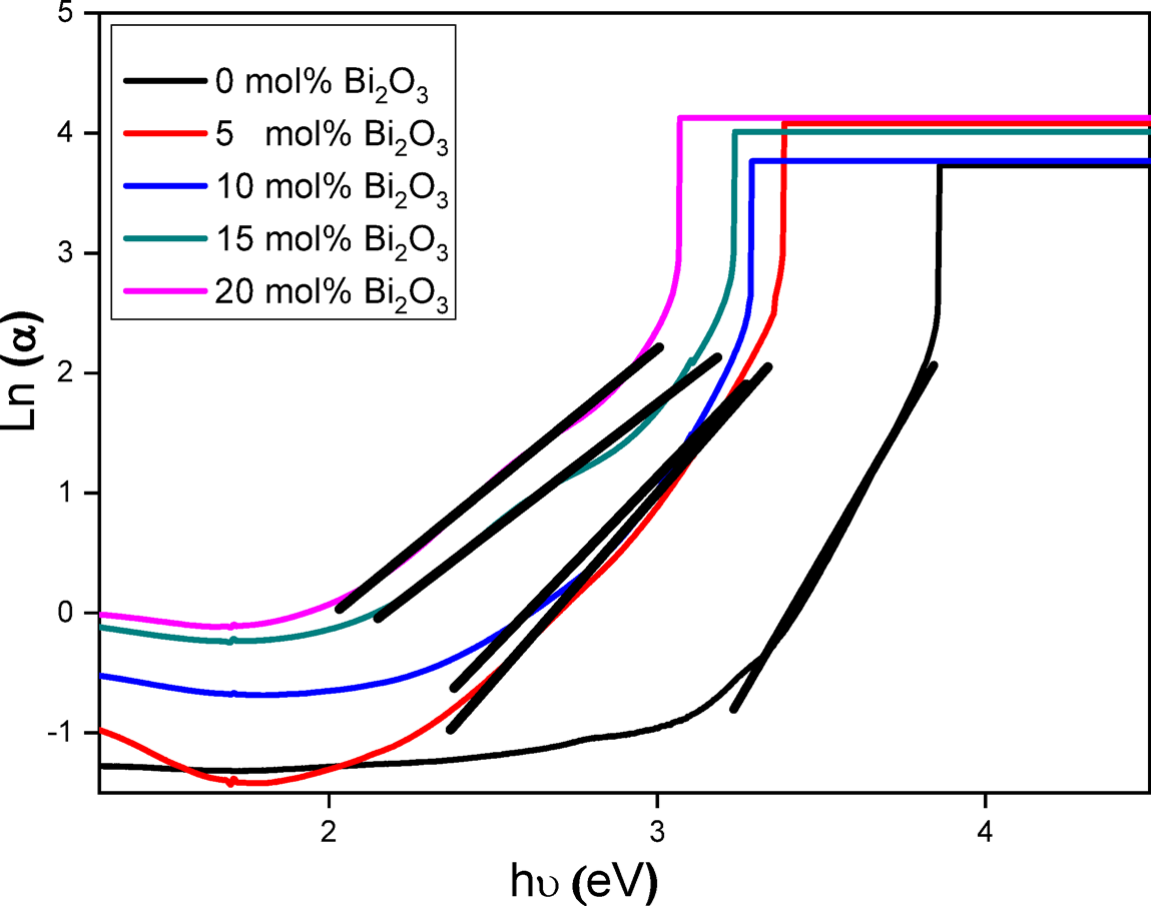
Urbach energy determination for the synthesized glasses, showing the natural logarithm of the absorption coefficient (ln α) against photon energy (hν).

**Fig. 9:**
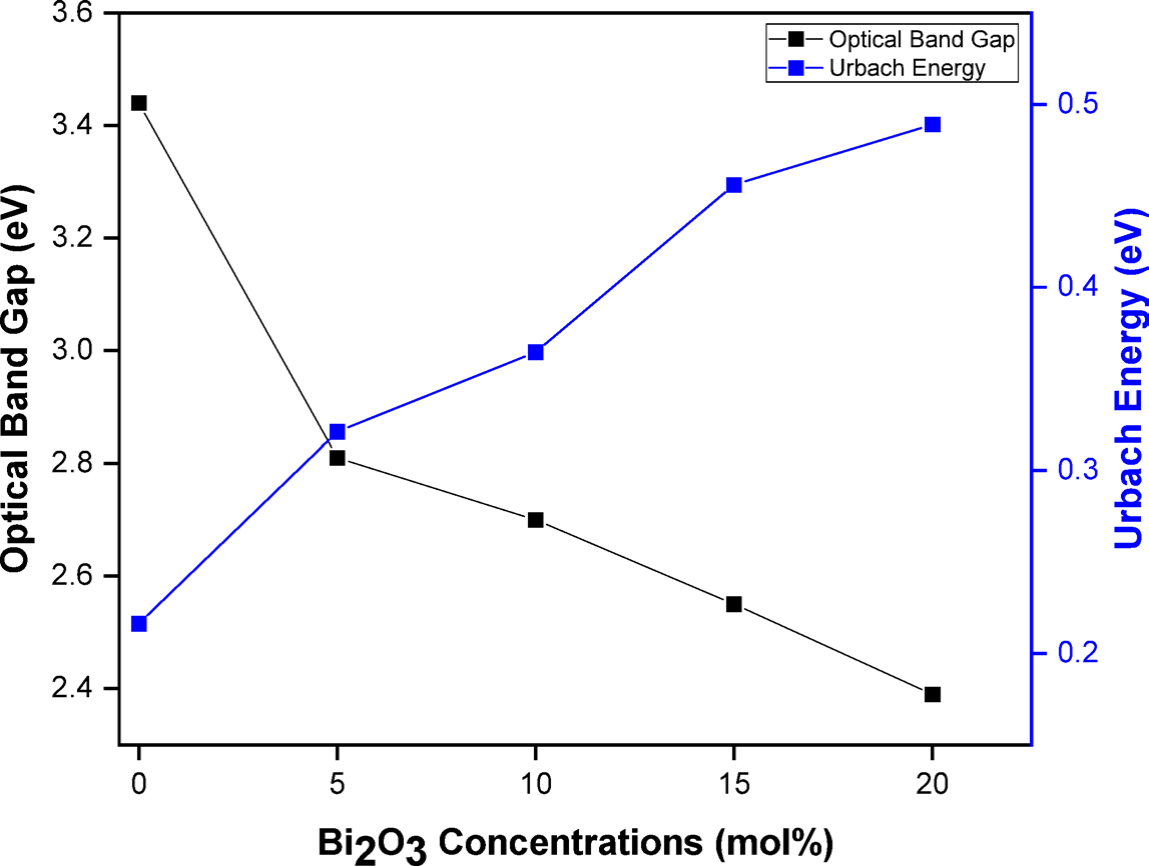
Variation of optical bandgap energy (E₉) and Urbach energy (E_u_) with Bi₂O₃ concentration.

As shown in Fig. 8; Table 4, the E_U_ values increased from **0.2164 eV** (lowest Bi₂O₃ content) to **0.488 eV** (highest Bi₂O₃ content). Notably, the trends for bandgap energy and Urbach tails were inversely correlated. The reduction in the optical bandgap may not reflect an actual decrease in bandgap energy but rather the influence of dopant-induced localized states. Consequently, samples with higher Bi₂O₃ content are more prone to defect formation, enhancing amorphous character, leading to increased Urbach energy and a reduced optical bandgap. The relationship between optical bandgap energy and Urbach tails as a function of Bi₂O₃ concentration is illustrated in Fig. 9.

### Gamma radiation attenuation properties

**Fig. 10:**
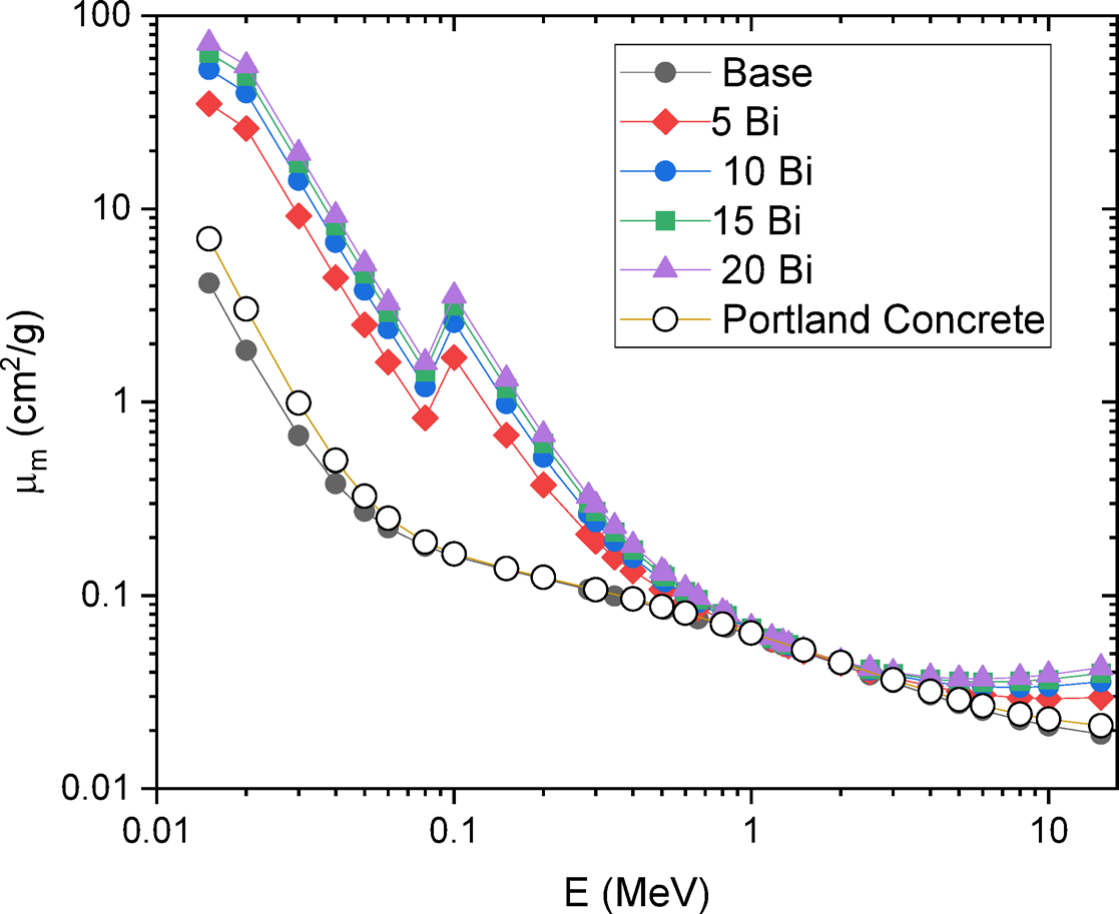
MAC of 30SiO2-(50-x) B2O3-5CaO-10Na2O-5LiF-xBi2O3 glasses where 20 ≥ x ≥ 0 mol%.

**Fig. 11:**
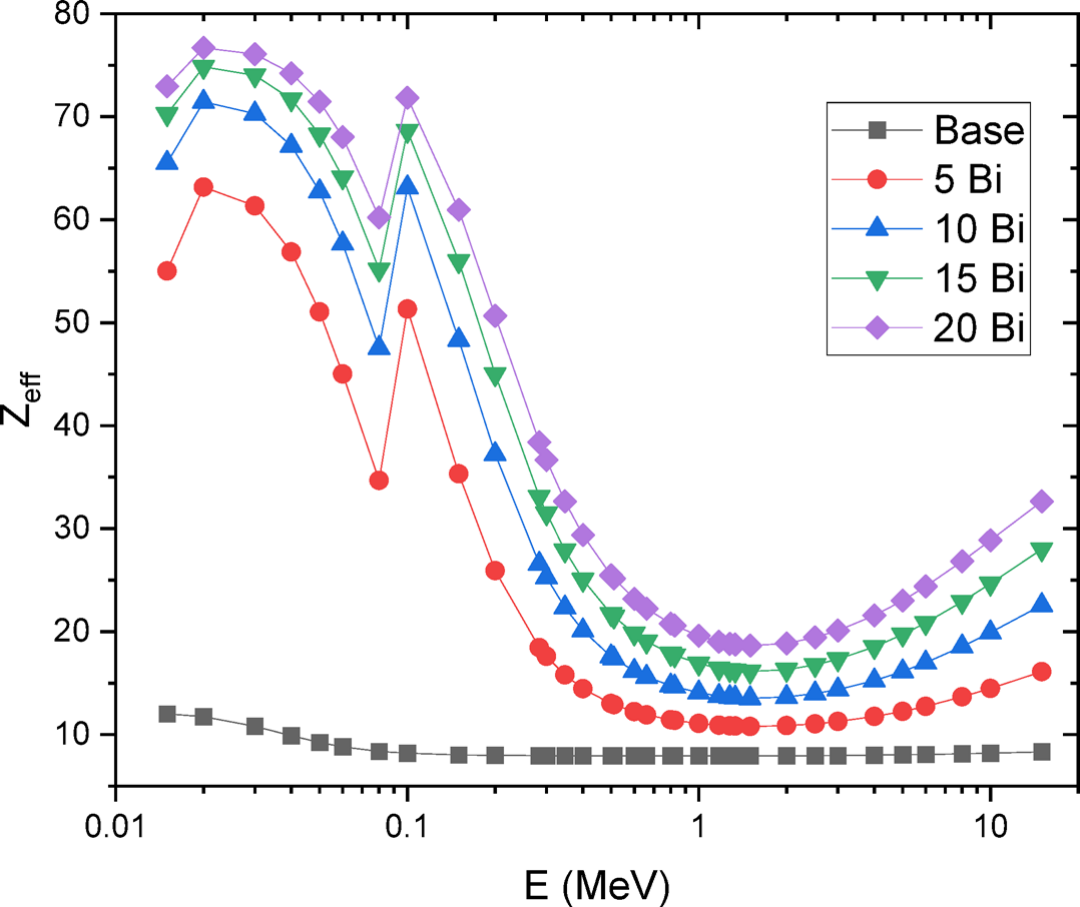
Effective atomic number of 30SiO2-(50-x) B2O3-5CaO-10Na2O-5LiF-xBi2O3 glasses where 20 ≥ x ≥ 0 mol%.

The mass attenuation coefficient (µ_m_), shown in Fig. 10, and the effective atomic number (Z_eff_), presented in Fig. 11, were evaluated for the manufactured borosilicate glass composites with varying Bi₂O₃ concentrations over the energy range of 0.015–15 MeV. The results show that µ_m_ decreases with increasing photon energy, while it increases with higher Bi₂O₃ content as boron is progressively replaced.

Figure 10 also highlights a marked enhancement in µ_m_ for all synthesized glass samples compared to Portland concrete, a commonly used radiation shielding material. The observed peak in µm around 0.1 MeV for the glass composition 30SiO₂-(50–x)B₂O₃−5CaO-10Na₂O-5LiF-xBi₂O₃ is attributed to intrinsic interactions between the glass samples and gamma radiation at this energy. At 0.04 MeV, gamma-ray interaction is dominated by the photoelectric effect, which strongly depends on both Z_eff_ and photon energy. The probability of photoelectric absorption increases with higher Z_eff_ and decreases with photon energy, following an approximate Z³/E³ relationship, where E denotes photon energy. The presence of high-Z elements such as bismuth (Z = 83) significantly enhances photon interaction through the photoelectric effect, particularly at lower energies.

Thus, the incorporation of Bi₂O₃ into the glass introduces high-Z elements that are highly effective at attenuating low-energy gamma rays via the photoelectric effect. This leads to an increased µ_m_ at 0.04 MeV, where strong interactions occur with the inner electron shells of Bi, further amplifying absorption.

From a radiation interaction perspective, substituting boron (B, Z = 5) with bismuth (Bi, Z = 83) leads to an increase in Z_eff_, thereby enhancing the shielding efficiencies of the glass. As Z_eff_ rises, the effectiveness of the material in attenuating gamma radiation also improves, making it a more efficient shielding medium.

.

**Fig. 12:**
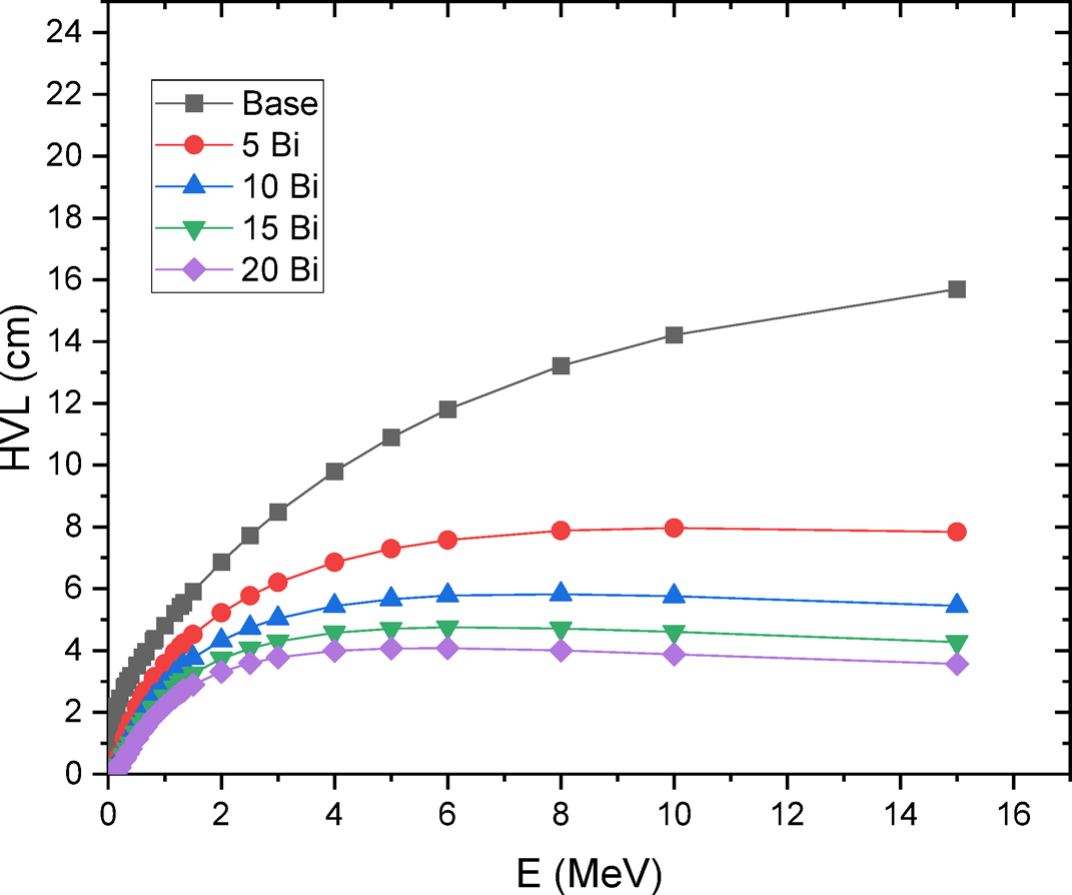
HVL of 30SiO2-(50-x) B2O3-5CaO-10Na2O-5LiF-xBi2O3 glasses where 20 ≥ x ≥ 0 mol%.

Figure 12 presents the half-value layer (HVL) measurements for the fabricated glass systems. The results indicate that HVL increases with rising photon energy and decreases as bismuth (Bi) concentration increases. Consequently, incorporating higher Bi content enhances gamma-ray attenuation.

A comparison between the theoretical and experimental values of the mass attenuation coefficient (µ_m_) at 0.662 MeV, as shown in Table 5, reveals a deviation of less than 10%. This suggests that computational methods can be effectively used to estimate attenuation parameters for various compositions before conducting costly experimental procedures.

**Table 5:** Experimental and theoretical results of values of µ_m_ of 30SiO_2_-(50-x)B_2_O_3_−5CaO-10Na_2_O-5LiF-xBi_2_O_3_ glasses where 20 ≥ x ≥ 0 mol%, at energies of 0.662 MeV.

	µ_m_ (cm^2^/g)	
Bi_2_O_3_ mol%	Experimental	Theoretical
0	0.0787 ± 0.0173	0.0757
5	0.0844 ± 0.0265	0.0860
10	0.0904 ± 0.0253	0.0918
15	0.1020 ± 0.0269	0.0956
20	0.1106 ± 0.0343	0.0983

**Fig. 13:**
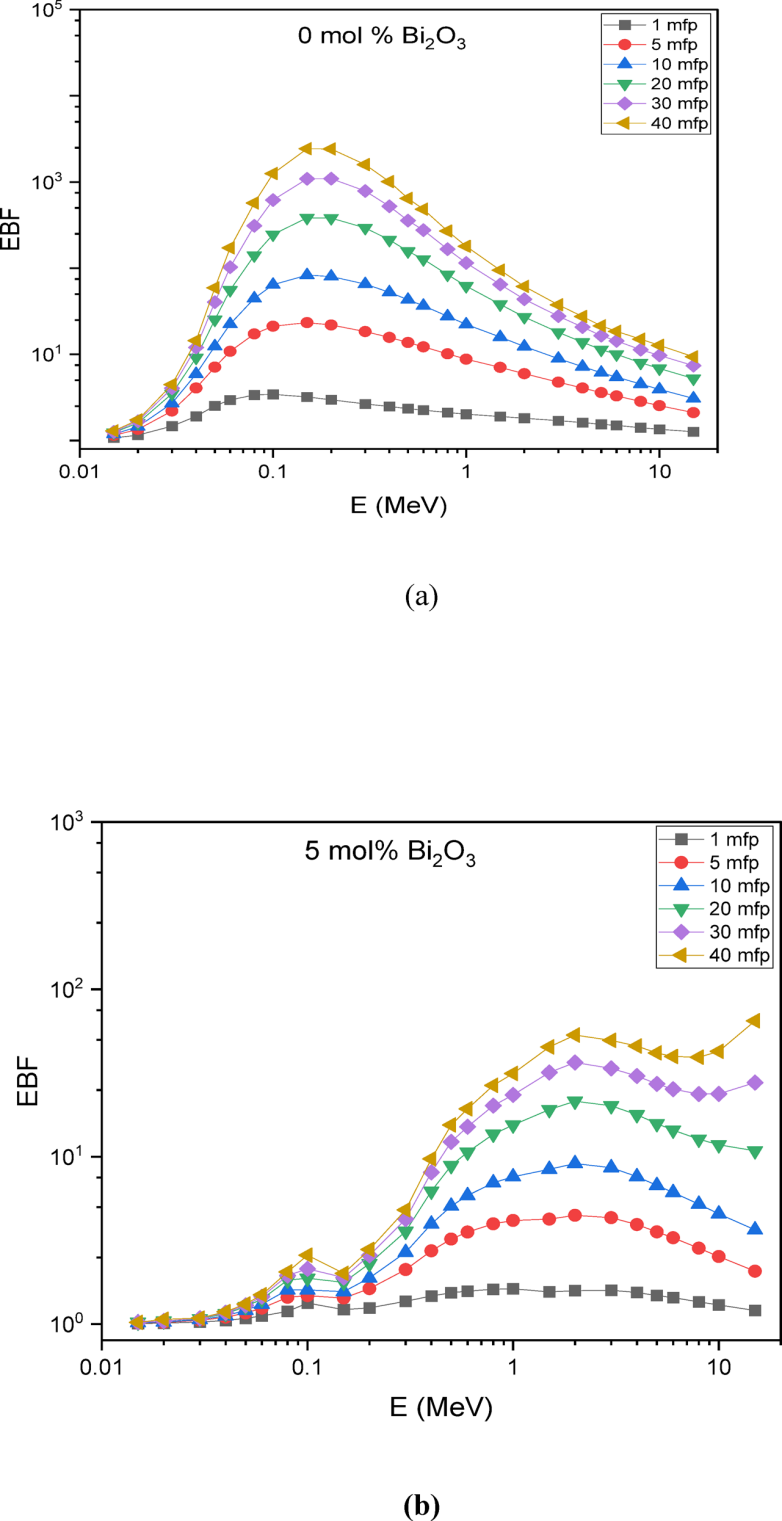

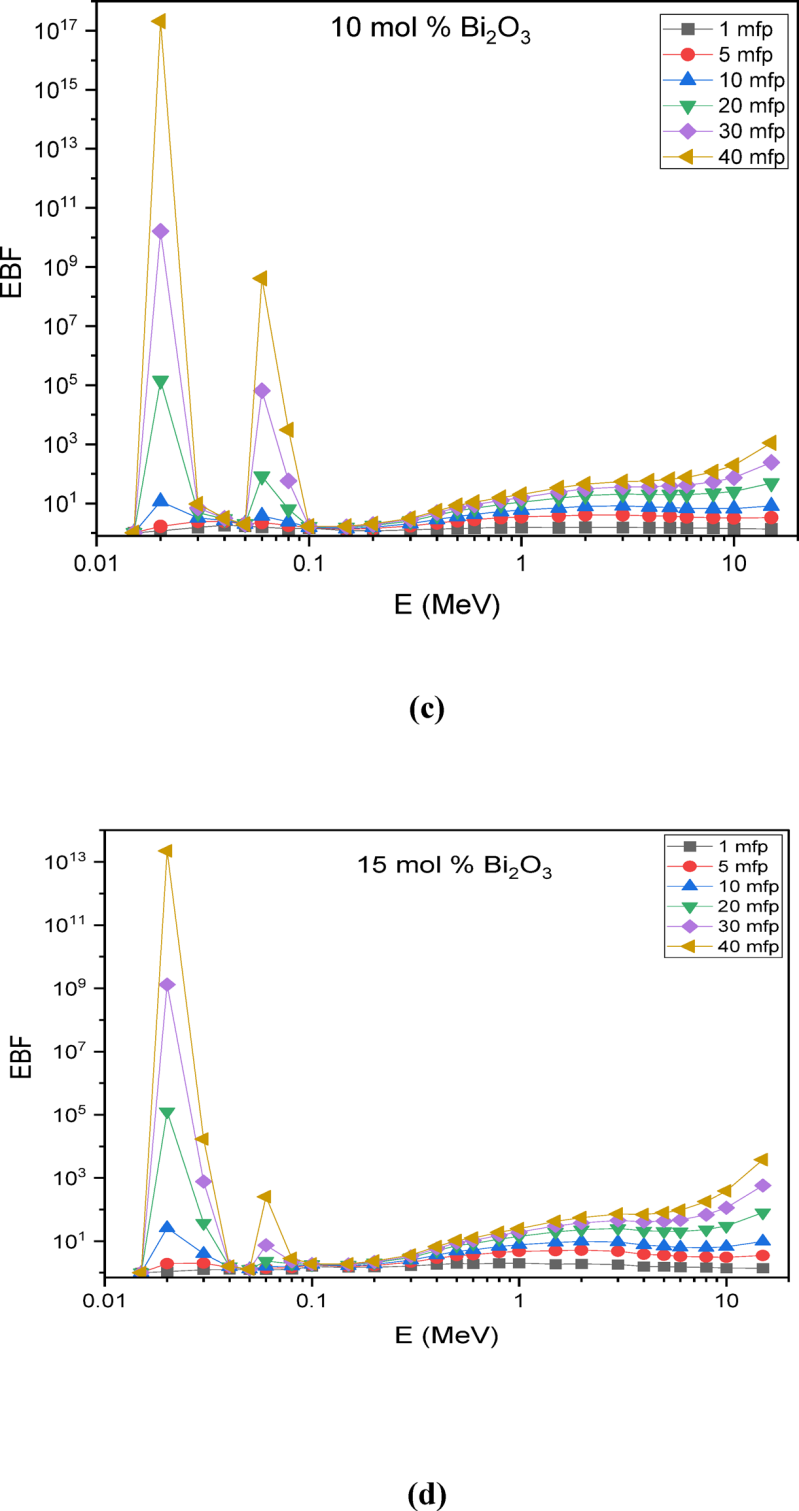

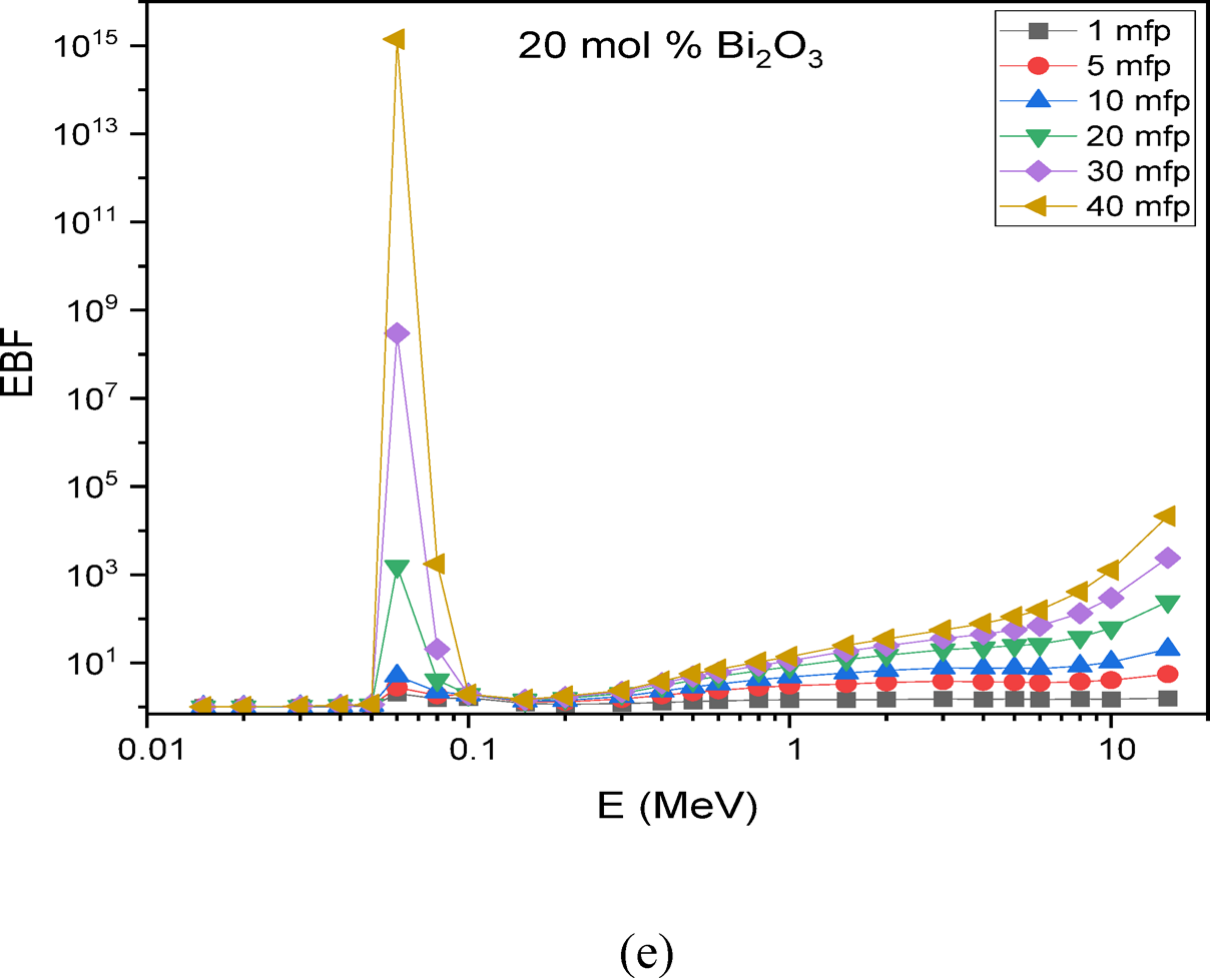
Exposure build-up factors for the prepared glass samples at photon energies from 0.015–15 MeV up to 40 mfp for (a) Base (0 mol % Bi_2_O_3_), (b) 5 mol % Bi_2_O_3_, (c) 10 mol % Bi_2_O_3_, (d) 15 mol % Bi_2_O_3_, (e) 20 mol % Bi_2_O_3_.

The Phy-X/PSD software was employed to compute the buildup factors of the glass systems^25^. Figure 13 illustrates the change in the Exposure Buildup Factor (EBF) with incident photon energy in the scale from 0.015 to 15 MeV. The results indicate that at low photon energies, the EBF values remain minimal, mainly because of the dominance of the photoelectric effect.

Notable peaks in the EBF are directly associated with various radiation-matter interactions, including Compton scattering, pair production, resonance absorption, and the photoelectric effect. These interactions vary significantly across different energy levels, resulting in certain energies where radiation accumulation becomes notably higher. Understanding these peaks is crucial for designing effective radiation shielding materials for diverse applications.

Specifically, the glass composition 30SiO₂-(50-x) B₂O₃−5CaO-10Na₂O-5LiF-xBi₂O₃ exhibits EBF peaks at ~ 0.03–0.04 MeV and at ~ 0.08–0.1 MeV. These peaks are attributed to the atomic and electronic structures of the material, which influence its interaction with gamma radiation at these specific energy levels.

These two peaks in the exposure buildup factor arise from different photon interaction mechanisms, explaining their contrasting behavior with increasing Bi₂O₃ content. The first peak, at low energies (~ 0.03–0.04 MeV), is dominated by the photoelectric effect, whose probability varies approximately as Z³/E³, making it highly sensitive to the effective atomic number (Z_eff_) of the glass^53^. Introducing Bi (Z = 83) increases Z_eff_ and enhances photon absorption, which suppresses multiple scattering and reduces buildup in this region. In contrast, the second peak (~ 0.08–0.1 MeV) falls within the Compton scattering domain, where the interaction cross-section depends primarily on electron density rather than Z_eff_^54^. Since electron density changes less significantly with Bi substitution compared to the sharp variation in photoelectric absorption, the magnitude of this peak remains relatively constant across different Bi₂O₃ concentrations.

Table 6 compares the fabricated glasses with reported data for barite concrete, Portland concrete, and lead-doped borate glasses. Although the synthesized glass samples contain neither lead nor barium, their effective atomic numbers and mass attenuation coefficients at 0.662 MeV are comparable to those of lead-rich glasses. Furthermore, all developed glasses demonstrate superior gamma-ray shielding performance compared to both Portland and barite concrete.

**Table 6:** Comparative analysis of different shielding materials at 662 keV.

System	Lead or barium concentration (mol %)	Rang of µm (cm2/g)x10−2	Z eff	Reference
Lead sodium borate glasses (PbO–Na 2 O–B 2 O 3)	Pb: 5–25	8.04–9.30	8.78–15.82	^55^
Lead borate glasses (PbO-B 2 O 3)	Pb: 30–70	8.31–9.96	9.96–21.25	^56^
Zinc sodium borate glasses (B 2 O 3 –Na 2 O–ZnO-Dy 2 O 3 -BaO)	Ba: 0–50	7.55–7.70	-	^57^
Lead sodium Lithium borosilicate glasses (PbO-Na 2 O-SiO 2 -B 2 O 3 -LiO 3)	Pb: 5–25	7.89–8.73	8.06–10.54	^58^
Barite concrete	-	6.7–7.8	-	^59,60^
Concrete Portland	-	7.76		^54^
Bismuth sodium lithium borosilicate glasses (SiO 2 -B 2 O 3 -CaO-Na 2 O-LiF-Bi 2 O 3)	Bi: 5–20	7.58–9.83	7.95–22.24	Current work

## Conclusion

This study demonstrates that incorporating Bi₂O₃ into lithium borosilicate glass significantly enhances both the optical response and radiation shielding efficiency. The glass samples maintained an amorphous structure across all compositions, with density increasing markedly from 2.31 g/cm³ (0 mol% Bi₂O₃) to 4.59 g/cm³ (20 mol% Bi₂O₃), alongside a molar volume expansion from 27.33 to 31.02 cm³/mol. FTIR analysis confirmed that Bi₂O₃ promotes the conversion of BO₃ to BO₄ units and increases non-bridging oxygens, consistent with observed optical modifications.

Optically, the fundamental band gap decreased from 3.44 eV to 2.39 eV, while the Urbach energy rose from 0.216 to 0.488 eV with increasing Bi₂O₃ content. This indicates higher structural disorder and greater electronic defect density, rendering these glasses suitable for optoelectronic and photonic applications requiring tailored absorption edges.

From a shielding perspective, the mass attenuation coefficient (µ_m_) increased with Bi₂O₃ loading, reaching 1.106 × 10⁻¹ cm²/g at 0.662 MeV for the 20 mol% Bi₂O₃ glass, substantially higher than that of Portland concrete (7.76 × 10⁻² cm²/g). Correspondingly, the effective atomic number (Z_eff_) rose to 22.24, and the half-value layer (HVL) decreased as Bi₂O₃ concentration increased, confirming superior attenuation performance. Importantly, the agreement between experimental and theoretical µm values (deviation < 10%) validates the reliability of predictive computational models. In comparison with conventional barite and Portland concretes, and even with certain lead-doped glasses, the Bi₂O₃-doped lithium borosilicate system achieved equal or superior shielding capabilities while remaining lead-free and environmentally benign.

Overall, the integration of Bi₂O₃ into lithium borosilicate glasses offers a dual advantage: tunable optical properties and enhanced gamma-ray shielding. These findings highlight their strong potential as transparent, non-toxic, and structurally stable alternatives for advanced optical devices, nuclear facilities, and medical radiation protection applications.

## Data Availability

Data is provided within the manuscript.
